# Functional and structural similarities of D7 proteins in the independently-evolved salivary secretions of sand flies and mosquitoes

**DOI:** 10.1038/s41598-019-41848-0

**Published:** 2019-03-29

**Authors:** Willy Jablonka, Il Hwan Kim, Patricia H. Alvarenga, Jesus G. Valenzuela, Jose´ M. C. Ribeiro, John F. Andersen

**Affiliations:** The Laboratory of Malaria and Vector Research, NIAID, National Institutes of Health, Rockville, Maryland 20852 USA

## Abstract

The habit of blood feeding evolved independently in many insect orders of families. Sand flies and mosquitoes belong to separate lineages of blood-feeding Diptera and are thus considered to have evolved the trait independently. Because of this, sand fly salivary proteins differ structurally from those of mosquitoes, and orthologous groups are nearly impossible to define. An exception is the long-form D7-like proteins that show conservation with their mosquito counterparts of numerous residues associated with the N-terminal domain binding pocket. In mosquitoes, this pocket is responsible for the scavenging of proinflammatory cysteinyl leukotrienes and thromboxanes at the feeding site. Here we show that long-form D7 proteins AGE83092 and ABI15936 from the sand fly species, *Phlebotomus papatasi* and *P*. *duboscqi*, respectively, inhibit the activation of platelets by collagen and the thromboxane A_2_ analog U46619. Using isothermal titration calorimetry, we also demonstrate direct binding of U46619 and cysteinyl leukotrienes C_4_, D_4_ and E_4_ to the *P*. *papatasi* protein. The crystal structure of *P*. *duboscqi* ABI15936 was determined and found to contain two domains oriented similarly to those of the mosquito proteins. The N-terminal domain contains an apparent eicosanoid binding pocket. The C-terminal domain is smaller in overall size than in the mosquito D7s and is missing some helical elements. Consequently, it does not contain an obvious internal binding pocket for small-molecule ligands that bind to many mosquito D7s. Structural similarities indicate that mosquito and sand fly D7 proteins have evolved from similar progenitors, but phylogenetics and differences in intron/exon structure suggest that they may have acquired the ability to bind vertebrate eicosanoids independently, indicating a convergent evolution scenario.

## Introduction

Salivary proteins in blood feeding arthropods are injected into the host feeding site where they prevent processes of hemostasis and inflammation, allowing the normal ingestion of blood^1^. Feeding is essential for the development of eggs and is also the route by which most vector-borne diseases are transmitted. Because of the intimate association between saliva and transmitted pathogens, they can be considered as a single entity and salivary proteins are currently being developed as vaccine candidates for vector-borne diseases^2–4^. The blood feeding habit has evolved numerous times in insects, and in each instance the evolution of essentially novel salivary proteins has occurred^5,6^. In the order Diptera (flies), blood feeding has appeared independently in two phylogenetic lineages within the suborder Nematocera^6^. The infraorder Culicomorpha represents one of these and contains the mosquitoes, black flies and biting midges. Concordant with the phylogeny of this group, the salivary secretions share orthologous proteins, and in several cases these proteins have been experimentally shown to function in the same manner. The second lineage of blood-feeding Nematocera consists of sand flies in the Phlebotominae, a subfamily of the family Psychodidae. Sand flies are vectors of parasites in the genus *Leishmania*, the causative agents of leishmaniasis, an important disease in the old and new world. Sand flies and the Culicomorpha differ in the families of proteins utilized for most salivary functions. For example, different structural families are employed as apyrases, anticoagulants, vasodilators and biogenic amine binding proteins in the two groups, reflective of their independent evolution^6^.

The D7 salivary proteins of mosquitoes, belong to the insect odorant-binding protein (OBP) family. They possess either one (short-form) or two (long-form) OBP domains and bind host-produced eicosanoid and biogenic amine compounds^7–9^. These small-molecule effectors are released in the skin in response to feeding where they mediate vascular tone, platelet activation and local inflammation. In *Aedes* and *Anopheles* mosquitoes, the two-domain D7 proteins bind eicosanoid ligands in a generally hydrophobic pocket of the N-terminal OBP domain. AeD7 the D7 from *Ae*. *aegypti* binds only cysteinyl leukotrienes in this pocket, while AnStD7L1, a homolog from *An*. *stephensi* binds both cysteinyl leukotrienes and thromboxane A_2_ (TXA_2_) in the corresponding pocket^8,9^. A single phenylalanine to tyrosine transition imparts the ability to bind TXA_2_ due to the formation of a new hydrogen bond interaction between the tyrosine hydroxyl group the ω-5 hydroxyl group of the thromboxane fatty acid^9^. Based on this sequence feature, it has been predicted that both anopheline and culicine mosquitoes contain two-domain D7 forms that bind cysteinyl leukotrienes and thromboxanes. AeD7 also binds the biogenic amines serotonin, norepinephrine and histamine at a site in its C-terminal domain. The C-terminal domain of AnstD7L1 is structurally similar but the binding site has been rearranged causing the protein to lose the ability to bind these compounds. Interestingly, the biogenic amine binding function in *Anopheles* appears to have been taken over by a group of short form D7 proteins whose genes are chromosomally linked to the long form protein^7^.

Despite the ‘rule of thumb’ that blood feeders from independent evolutionary lineages utilize different protein families for various salivary functions, the saliva of sand flies contains apparent homologs of the two-domain long-form D7 proteins of mosquitoes^10,11^. The similarity is particularly strong in the predicted N-terminal domain of the protein where key amino acids lining the leukotriene/thromboxane binding pocket that interact with the ligand are conserved. In this study we demonstrate, using binding studies and bioassays, that the sand fly long-form D7s do in fact bind both cysteinyl leukotrienes and thromboxanes, and thereby serve as anti-inflammatory and anti-hemostatic agents in both sandflies and mosquitoes. We also describe the crystal structure of the sand fly protein where we examine the conserved N-terminal domain, as well as the more divergent C-terminal domain and domain interface region to assess relatedness with the mosquito D7 forms and the evolutionary origins of the group as a whole.

## Results

**Figure 1:**
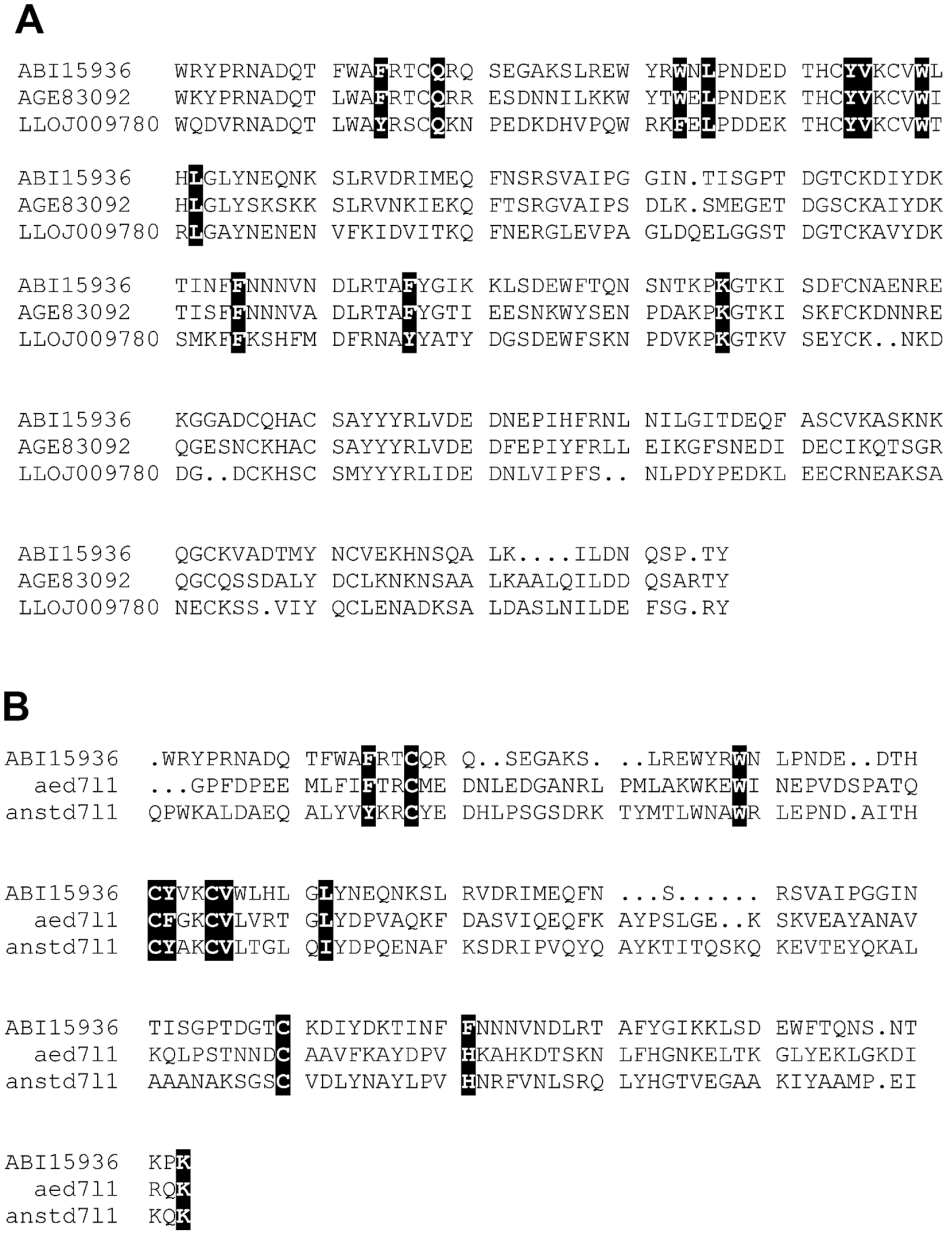
Sequences of sand fly D7 proteins. (**A**) Comparison of long-form D7 sequences from *Phlebotomus duboscqi* (ABI15936), *P*. *papatasi* (AGE83092) and *Lutozomyia longipalpus* (LLOJ009780). Key residues contained in the N-terminal domain ligand binding pocket are highlighted in black. (**B**) Comparison of the N-terminal domain sequence from ABI15936 with those of AeD7 from *Aedes aegypti*, and AnStD7L1 from *Anopheles stephensi*. The highlighted residues are known to contact bound eicosanoid ligands in the crystal structures of protein ligand complexes (AeD7 PDB ID 3DZT, AnStD7L1 PDB ID 3NHT).

Long-form D7 proteins are found in the salivary secretions of *Phlebotomus* and *Lutzomyia* sand flies from the new world and old world respectively (Fig. 1A). Sequence comparisons suggest that many of the residues involved in the binding of ligands in the N-terminal domain of the protein are conserved with apparent homologs in the saliva of mosquitoes including AeD7 from *Ae*. *aegypti* and AnStD7L1 from *An*. *stephensi* (Fig. 1A,B). In the genome of *Phlebotomus papatasi*, AGE83092 and AGE83093, a pair of related long-form D7s are separated by 6.7 kb on the chromosome indicating that they are the result of a tandem duplication event^12^. *P*. *papatasi* is distributed throughout the Mediterranean region and the Middle East where it acts as a major vector of *Leishmania*. In sub-Saharan Africa, a closely related sand fly species, *P*. *duboscqi*, serves as the major vector in this region. Although the *P*. *duboscqi* genome has not been sequenced, long-form D7 transcripts have been identified in salivary cDNA libraries from this species^13^. In the new world, members of a second sand fly genus, *Lutzomyia*, are responsible for transmission of *Leishmania*, and contain long-form D7 proteins in their saliva (Fig. 1A)^14^. Previous studies have shown that the residues involved with eicosanoid ligand binding in the putative N-terminal domain of the two-domain long-form D7 proteins from mosquitoes are conserved in the apparently homologous proteins of sand flies, but residues associated with biogenic amine binding in the C-terminal domain of the long-form D7 proteins of *Aedes* species are not (Fig. 1B)^6,10^. We produced recombinant versions of two representative forms of sand fly long-form D7 from *P*. *papatasi* (AGE83092) and *P*. *duboscqi* (ABI15936) and evaluated their structure, ligand-binding properties and physiological function using X-ray diffraction, isothermal titration calorimetry (ITC), and bioassay with human platelets.

### Ligand binding by long-form D7s from sand flies

**Figure 2:**
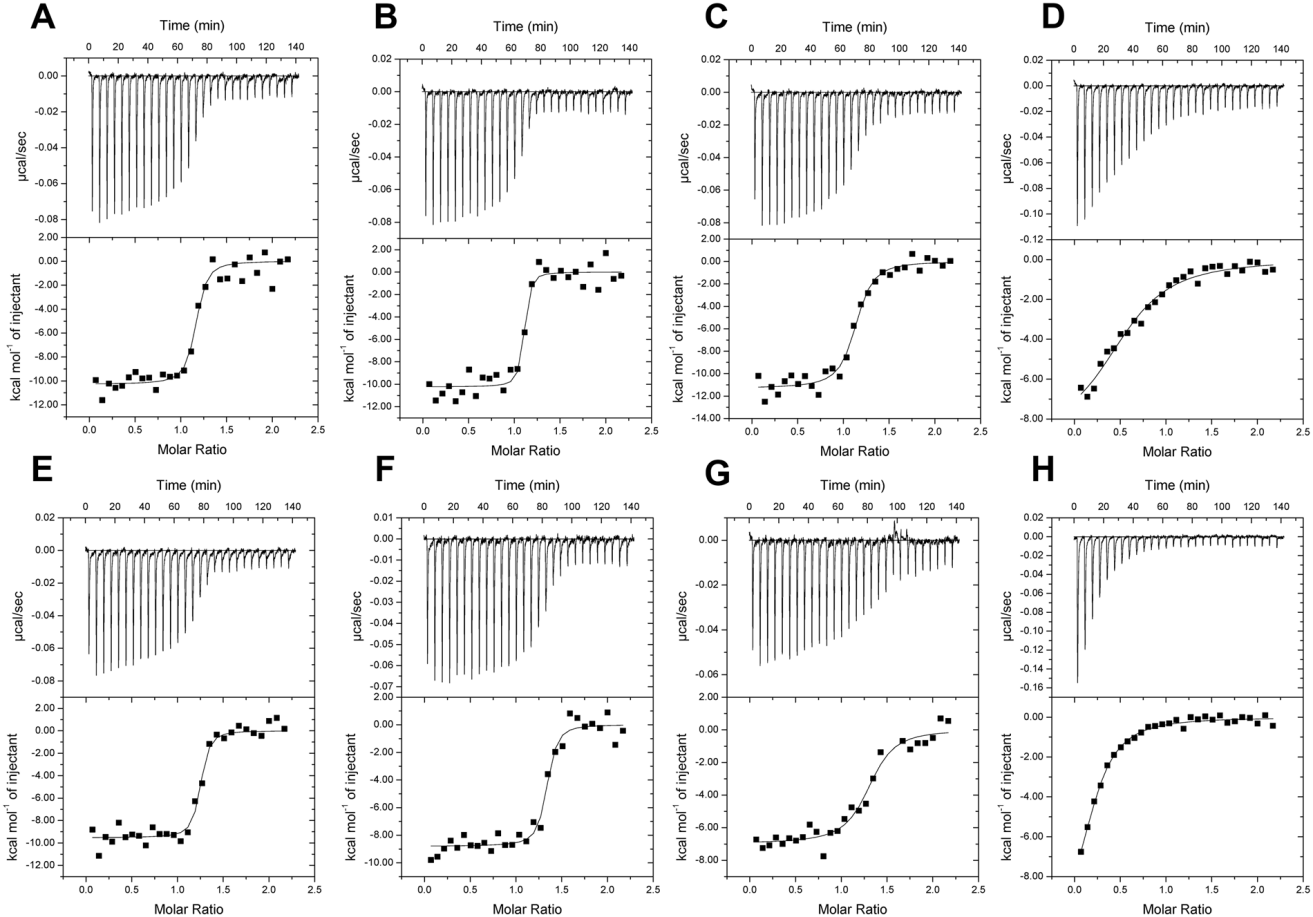
ITC analysis of eicosanoid binding. For cysteinyl leukotriene experiments, the calorimeter cell was filled with *P*. *papatasi* D7 protein AGE83092 (**A**–**C**) or *P*. *duboscqi* ABI15936 (**E**–**G**) at 2 μM in Tris-buffered saline. The syringe contained 20 μM LTC_4_ (**A**,**E**), LTD_4_ (**B**,**F**) and LTE_4_ (**C**,**G**) in the same buffer. Injections (10 µL) were spaced at 300 s intervals. For U46619 (**D**,**H**), the protein concentration in the cell was 5 µM for AGE83092 (**D**) and 10 µM for ABI15936 (**H**). The syringe concentration was 50 µM AGE83092 and 100 µM for ABI15936. Heats were recorded on a VP-ITC MicroCalorimeter and data were fit using a single binding site model on the MicroCal software package (Origin 7) for calculation of thermodynamic parameters. Titration curves are representative of at least 3 measurements. Thermodynamic parameters: Panel A K_a_ = 1.7 × 10^8^ M^−1^, ΔH = −10.3 kcal/mol, N = 1.1 sites; Panel B K_a_ = 5.7 × 10^8^ M^−1^, ΔH = −10.2 kcal/mol, N = 1.1 sites; Panel C K_a_ = 6.3 × 10^7^ M^−1^, ΔH = −11.3 kcal/mol, N = 1.1 sites; Panel D K_a_ = 1.33 × 10^6^ M^−1^, ΔH = −8.5 kcal/mol, N = 0.6 sites; Panel E K_a_ = 3.1 × 10^8^ M^−1^, ΔH = −8.6 kcal/mol, N = 1.3 sites; Panel F K_a_ = 2.4 × 10^8^ M^−1^, ΔH = −8.9 kcal/mol, N = 1.3 sites; Panel G K_a_ = 3.4 × 10^7^ M^−1^, ΔH = −7.0 kcal/mol, N = 1.3 sites; Panel H K_a_ = 7.8 × 10^5^ M^−1^, ΔH = −11.5 kcal/mol, N = 0.25 sites.

We used ITC to examine the binding affinities of *P*. *papatasi* AGE83092 and *P*. *duboscqi* ABI15936 for the known ligands of mosquito D7 proteins, including biogenic amines, cysteinyl leukotrienes, and the thromboxane A_2_ (TXA_2_) analog U46619. The proteins were found to bind a single molecule of the cysteinyl leukotrienes LTC_4_, LTD_4_ and LTE_4_ (Fig. 2). The three ligands bound similarly, with LTC_4_ and LTD_4_ showing affinities (1/K_a_) of 2–6 nM, while LTE_4_ bound with a slightly lower affinity of 16–29 nM (Fig. 2). U46619 also proved to be a ligand for the D7 proteins exhibiting affinity constants of approximately 750 nM in AGE83092 and 1.3 µM in ABI15936, with apparently a single molecule binding per molecule of protein as in the mosquito homologs (Fig. 2). The proteins did not bind the biogenic amines histamine or serotonin, consistent with the lack of binding pocket conservation relative to AeD7 from *Ae*. *aegypti* or the single domain short-form D7s from *An*. *gambiae* (Fig. S1).

### Inhibition of platelet aggregation by sand fly D7s

**Figure 3:**
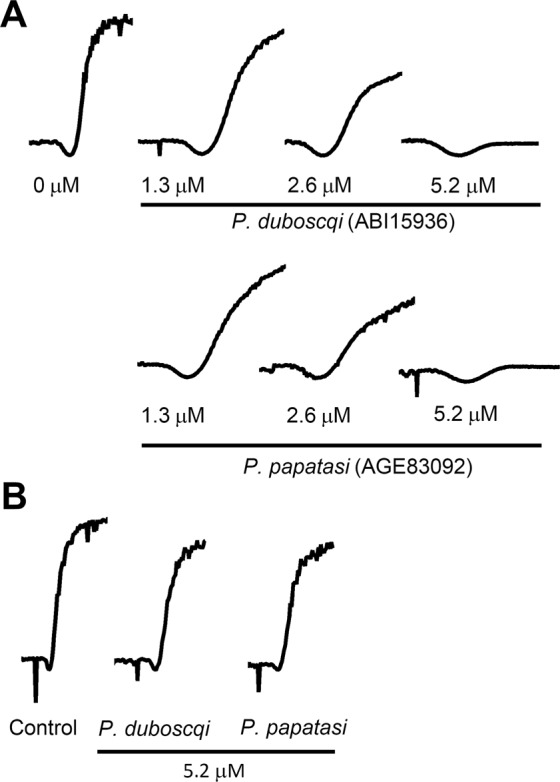
Sand fly D7s inhibit collagen-induced platelet aggregation. 10 mM Tris buffer pH 7.4 (control) or different concentrations (indicated on figure) of *P*. *dubosqi* ABI15936 or *P*. *papatasi* AGE83092 in Tyrode buffer were added to a stirring platelet suspension at 37 °C and incubated for 1 min in the aggregometer. Platelet aggregation was observed as an increase in transmittance (rise of the baseline) after addition of collagen at concentrations of 1.4 μg/ mL (low concentration) (**A**) or 14 μg/ ml (high concentration) (**B**). Traces are representative of three experiments performed in duplicate.

On contact with collagen exposed by wounding, quiescent platelets release granules and begin secreting TXA_2_. The granule component ADP along with secreted TXA_2_ potentiate the activation of platelets through specific G protein-coupled receptors, making them secondary agonists that are necessary for a robust platelet activation response. AnStD7L1, a long-form D7 from *An*. *stephensi* saliva has been shown to inhibit collagen- and U46619-mediated platelet activation indicating that it has the potential to block aggregation of platelets at the site of feeding^9^. A convincing case was made that this is due to direct binding and sequestration of the agonist TXA_2_. The aggregation of platelets in platelet-rich plasma was examined to determine if ABI15936 and AGE83092 exhibit similar inhibitory properties. We measured the activation and aggregation of platelet suspensions after exposure to 1.4 µg/mL collagen in the presence and absence of ABI15936 and AGE83092 (Fig. 3A). Collagen alone initiated a complete aggregation response as indicated by an increase in transmittance of dilute platelet suspensions, following a smaller decrease in transmittance due to platelet shape change (Fig. 3A). Addition of either AGE83092 or ABI15936 prior the collagen stimulus resulted in a concentration-dependent reduction in aggregation. Inhibition of aggregation was detectable at protein concentrations as low as 1.3 µM, and the inhibition was essentially complete at a concentration of 5.2 µM (Fig. 3A). At higher collagen concentrations, signaling through integrin receptors and platelet glycoprotein VI is sufficient to induce platelet aggregation without involvement of TXA_2_. We found that inhibition of platelet activation by either AGE83092 or ABI15936 was negligible at inhibitor concentrations of 5.2 µM when a collagen stimulus of 14 µg/mL was used (Fig. 3B). Inhibition of collagen-mediated aggregation at 1.4 µg/mL collagen, but not 14 µg/mL, is consistent with a mechanism of action involving TXA_2_ binding.

**Figure 4:**
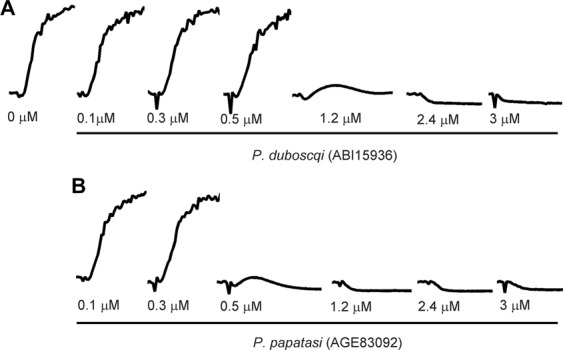
Sand fly D7s inhibit U46619-induced platelet aggregation. Stirred platelets were incubated with 10 mM Tris buffer pH 7.4 (control) or buffer containing *P*. *duboscqi* ABI159356 or *P*. *papatasi* AGE83092 D7 proteins (at the concentrations indicated on the figure) for one minute. Platelet aggregation was induced by addition of U46619 at a final concentration of 0.5 μM and monitored as described in Fig. 3. Traces are representative of three experiments performed in duplicate.

To further establish the role of sand fly D7s as TXA_2_-binding proteins, we tested the effect of these on platelet aggregation induced by the TXA_2_ analog U46619. Complete aggregation was seen after addition of 0.5 µM U46619 to a suspension of stirred platelets (Fig. 4). If either ABI15936 or AGE83092 were added and incubated with the platelet suspension prior to addition of agonist, the aggregation response was inhibited in a concentration-dependent manner (Fig. 4). Unlike the effects seen after collagen stimulation, inhibition appeared abruptly at concentrations at or above 0.5 µM protein. This is indicative of a sequestration mechanism, where the sequestering agent, in this case the D7 protein, needs to be present at a concentration equal to or higher than that of the ligand (Fig. 4). The effect is particularly pronounced when the concentration of ligand is near or above its dissociation constant. Taken together, experiments with collagen and U46619 indicate that the sand fly D7 proteins act as platelet aggregation inhibitors that function by sequestering TXA_2_. The lability of TXA_2_ prevents direct measurement of its binding to the D7 proteins, but the potency of the protein in collagen-mediated aggregation assays strongly suggests that the proteins bind natural TXA_2_ with similar or higher affinity than the analog U46619, as was previously seen with the *An*. *stephensi* D7 AnStD7L1.

### The crystal structure of *P*. *duboscqi* ABI15936

**Figure 5:**
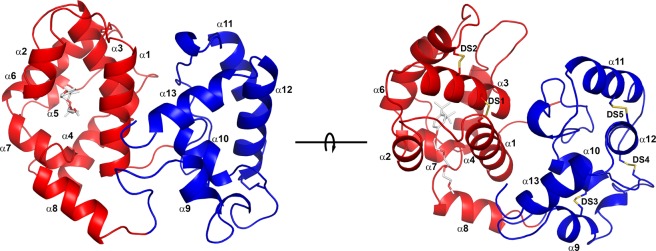
Ribbon diagram of *P*. *duboscqi* ABI15936. The models on the left and right are related by 90° rotation around the axis shown. The N-terminal domain is colored in red and the C-terminal domain in blue. Alpha helical segments are labeled α1-α13, and disulfide bonds are shown in stick representation in the right-hand figure and labeled DS1-DS5. Sulfur atoms are colored in yellow. The ligand Triton X-100 is also shown in the N-terminal binding pocket, in stick representation with carbon atoms colored light grey and oxygen colored red.

The structure of ABI15936 was determined using single anomalous diffraction methods with a selenomethionine-substituted variant protein prepared in *E*. *coli* (Table 1). An initial model containing two protein monomers was built using the experimental electron density map and then positioned into a wild-type data set by molecular replacement. The model was then completed by iterative cycles of rebuilding and refinement. As suggested by sequence comparisons, the protein is made up of two linked odorant binding protein (OBP) domains with a well-ordered domain interface fixing their relative orientations (Fig. 5). The N-terminal domain contains a hydrophobic channel leading from the surface to the interior of the protein that is much like the eicosanoid binding pocket in *Aedes* and *Anopheles* long-form D7 proteins. The C-terminal domain appears generally like those of the mosquito proteins but does not contain the biogenic amine binding pocket seen in AeD7, the *Ae*. *aegypti* long D7 protein that binds both eicosanoids and biogenic amines (Fig. 5).

**Table 1:** Data collection and refinement statistics for the *P*. *duboscqi* ABI15936.

Crystal	Selenium	Wild-type
Resolution (Å)	50.0–1.78	41.5–1.92
Beamline	22-ID	22-BM
Wavelength (Å)	0.97499	0.97910
Completeness (total/high resolution shell)	99.8/99.0	99.9/98.7
Average Redundancy (total/high resolution shell)	4.5/3.4	11.85/8.15
R_merge_ (total/high resolution shell, %)	6.5/19.4	7.8/36.4
CC_1/2_ (total/high resolution shell)	99.8/96.3	0.99/0.93
I/sigI (total/high resolution shell)	23.1/5.6	28.2/6.3
Observed reflections	387,747	459,266
Unique Reflections	46,308	38,751
Space group	P2_1_2_1_2	P2_1_2_1_2
Unit cell dimensions (Å)		
a	143.87	143.89
b	101.48	101.56
c	33.53	33.59
α, β, γ (°)	90	90
No. of selenium sites	6	
FOM (Phenix autosol)	0.35	
Bayes-CC	32.7	
Refinement		
Total non-H protein atoms	3751	3746
Total non-H solvent atoms	510	470
Total non-H ligand atoms	50	50
RMS deviations		
bond lengths (Å)	0.006	0.006
bond angles (°)	0.89	0.88
Wilson B factor (Å^2^)	13.7	14.8
Mean B factors (Å^2^)		
protein	15.7	16.2
solvent	26.7	25.0
ligand	18.0	13.1
Molprobity analysis		
Ramachandran plot (favored/allowed, %)	97.3/100	97.8/100
Clashscore	2.7	2.8
Rotamer outliers (%)	0.0	0.0
Coordinate error ML (Å, Phenix)	0.16	0.19
R_cryst/_R_free_	0.17/0.21	0.18/0.21

**Figure 6:**
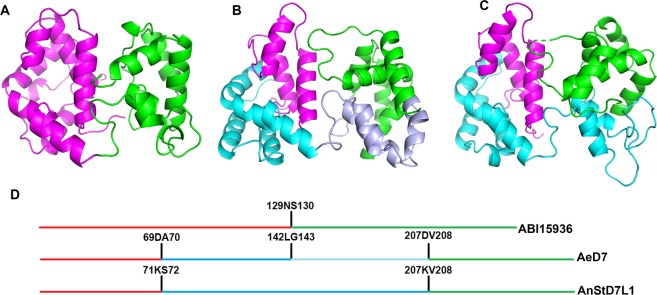
Salivary long-form D7 proteins from *P*. *duboscqi*, *An*. *stephensi* and *Ae. aegypti* colored according to exon structure. (**A**) *P*. *duboscqi* ABI15936 with the region encoded by exon 2 colored magenta and encompassing the entire N-terminal domain. The region encoded by exon 3 is colored green and includes the entire C-terminal domain. (**B**) *Ae*. *aegypti* AeD7 (3DYE) showing the N-terminal domain split into regions encoded by exon 2 (magenta) and exon 3 (cyan). The C-terminal domain is encoded by exons 4 (light blue) and 5 (green). (**C**) Like the *Aedes* protein, AnStD7L1 from *An*. *stephensi* (3NHT) has the C-terminal domain encoded by exons 2 (magenta) and 3 (cyan) but in this case exon 3 also encodes the first part of the C-terminal domain encoded by exon 4 in AeD7. The region encoded by exon 4 (green) corresponds to exon 5 of AeD7. (**D**) Schematic of the exon structure of sand fly and mosquito long-form D7 proteins. Horizontal lines represent the primary amino acid sequences of the coding regions color coded as in (**A**–**C**). The vertical lines represent the intron/exon boundaries with the amino acids at the joining sites listed.

The N-terminal domain of ABI15936 contains two disulfide bonds that are conserved in the mosquito proteins. These link Cys 17 with Cys 47 and Cys 45 with Cys 93 (Fig. 5). The C-terminal domain contains three disulfides linking Cys 143 with Cys 159, Cys 155 with Cys 202 and Cys 192 with Cys 211 (Fig. 5). Like the mosquito long D7 proteins the N-terminal domain is made up of seven α-helical segments (labeled α1-7, Fig. 5). Segment α5 is shortened to a single turn in the sand fly protein and is partially replaced by an extended coil region connecting α5 and α6. The segment connecting the N-terminal and C-terminal domains is essentially identical in conformation to the long-form D7 proteins from mosquitoes. The relative orientation of the C-terminal and N-terminal domains to one another is also similar in the mosquito and sand fly proteins, but the positioning of the four helical segments (α10-α13) in the C-terminal domain is somewhat different than that of the mosquito proteins. The genomic sequences of AGE83092 and AGE83093 from *P*. *papatasi* show the presence of three exons in each gene. Exon 1 encodes a signal peptide, while exons 2 and 3 encode the N- and C-terminal domains, respectively, with the exon-intron boundary located in the linker region between the two domains (Fig. 6A,D). The salivary D7 AeD7 from *Ae*. *aegypti* contains five exons with exon 1 encoding the signal peptide (Fig. 6B,D). The N- and C-terminal domains are encoded by two exons each and a third intron is present in the region encoding the domain-linking sequence (Fig. 6B). AnStD7L1 from *An*. *stephensi* shows conservation of the intron positions in the N- and C-terminal domains, but lacks the intron in the domain linker region, giving a total of four exons with one encoding the signal peptide (Fig. 6C,D).

### Binding pocket of the N-terminal domain

**Figure 7:**
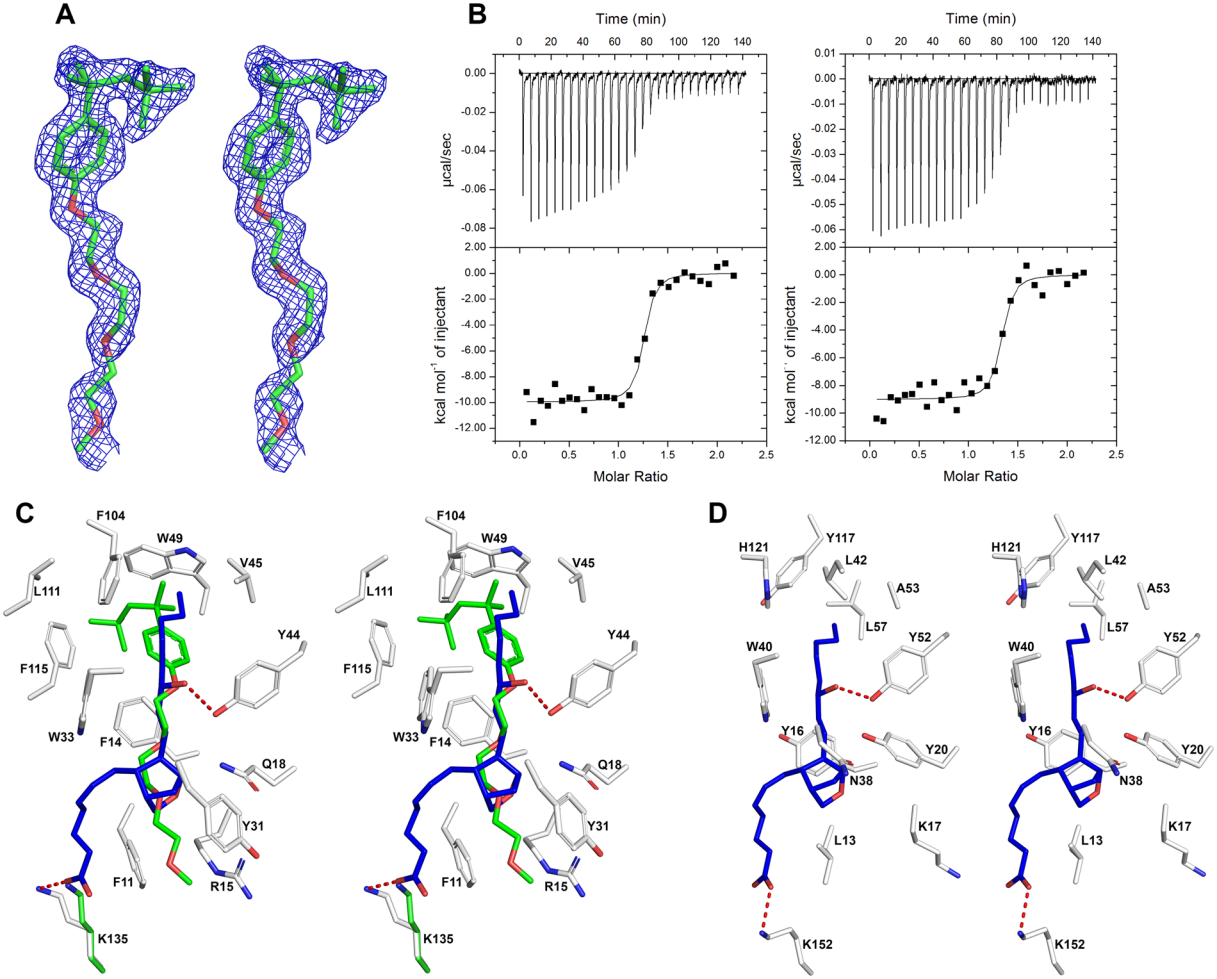
Binding pocket interactions in ABI15936. (**A**) Stereoview of bound Triton X-100 with weighted omit electron density (Fo-Fc) contoured at 3 σ covering the ligand. Carbon atoms are colored green and oxygen atoms are colored red (**B**). Comparison of LTC_4_ binding with ABI15936 prepared with (left, also shown in Fig. 2E) and without (right) Triton-X100 as measured by ITC using the same methods as in Fig. 2. Thermodynamic parameters without Triton X-100, K_a_ = 2.2 × 10^8^ M^−1^, ΔH = −10.0 kcal/mol, N = 1.2 sites. (**C**) Stereoview showing interactions of Triton X-100 with binding pocket residues of *P*. *duboscqi* ABI15936, also containing a modeled U46619 molecule positioned by superposition of the *An*. *stephensi* AnStD7L1-U46619 complex (PDB ID 3NHT). Triton X-100 atoms are colored as in panel A. In U46619, carbon is colored blue and oxygen red. In the protein, carbon is colored light grey, oxygen is colored red and nitrogen is colored blue. Potential hydrogen bonding interactions in the modeled U46619 complex are shown as dashed red lines. For modeling of the U46619 complex, only the position of the Lys 135 side chain was changed to avoid a steric clash. The refined position is shown in green and the rotated position in white. (**D**) Stereoview showing U46619 in the binding pocket of AnStD7L1 from *An*. *stephensi* (PDB ID 3NHT). Coloring of U46619 atoms, protein atoms and hydrogen bonds are as in panel C.

Although ABI15936 was crystallized in the absence of any potential natural ligands, electron density was observed indicative of a non-protein molecule occupying the N-terminal domain binding site. This was determined after refinement of the protein complex to be the detergent Triton X-100, which was retained through the steps of protein purification (Figs 5, 7A,C). A second sample of protein was prepared in the absence of detergent, and the binding properties of the two samples were compared using LTC_4_ as a ligand. Little difference in the equilibrium constant or other thermodynamic parameters was observed, indicating that Triton-X100 binding is not of sufficient affinity to exclude the host ligand at the micromolar concentrations used in the calorimetry experiments (Fig. 7B). We were unable to produce co-crystals of either the U46619 or LTC_4_ complexes under the same conditions as the ligand-free protein and when crystals were soaked with cryoprotectant solution containing LTC_4_ the resulting ligand electron density was not easily interpretable, suggesting a mixture of Triton-X100 and LTC_4_ complexes, with the Triton-X100 complex predominating. Perhaps elevated detergent concentrations in the crystal or steric constraints in the crystal prevented efficient ligand exchange during the soak period.

The binding pocket of ABI15936 is much like that of the mosquito long-form D7 proteins, with numerous residues important for ligand binding being conserved. The side chain of Lys 135 is oriented nearly identically to that of Lys 152 of AnStD7L1 where it is positioned to form a salt bridge with the carboxyl group of U46619 modeled into the pocket in the same conformation as in the crystal structure of the U46619-AnStD7L1 complex (Fig. 7C)^9^. The interior region of the pocket that accommodates the hydrophobic non-functionalized end of the eicosanoid is also highly conserved. Trp 35, Phe 14, Tyr 44 and Phe 115 are positioned much like their aromatic counterparts in AnStD7L1, and the aliphatic hydrophobic residues Leu 35, Val 48 and Leu 52 are also conserved in mosquito proteins (Fig. 7C,D). In previous studies, AeD7 from *Ae*. *aegypti* showed no affinity for U46619, while AnStD7L1 from *An*. *stephensi* bound this compound and was a potent platelet aggregation inhibitor^8,9^. A single phenylalanine to tyrosine change was responsible for the ability to bind TXA_2_ since the tyrosine hydroxyl group forms an essential hydrogen bond with the hydroxyl group at ω-5 position of the fatty acid. Cysteinyl leukotrienes are not hydroxylated at this position and bind similarly to either protein. Both ABI15936 and AGE83092 contain tyrosine at this position, and as expected, both inhibit platelet aggregation as shown above. In the structure of ABI15936, Tyr 44 is oriented identically to the homologous residue Tyr 52 in AnStD7L1 and can be assumed to play the same role in binding (Fig. 7C,D).

### Comparison of sand fly and mosquito C-terminal domains

**Figure 8:**
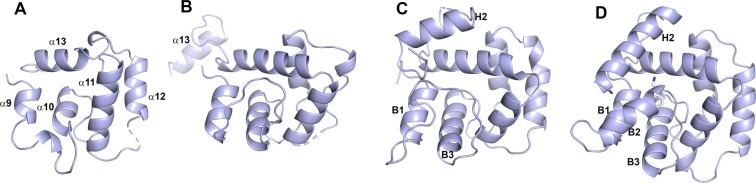
C-terminal domain structures of long-form D7 and related proteins. Models of the C-terminal domains of *P*. *duboscqi* ABI15936 (**A**), hemolymph mJHBP from *Ae*. *aegypti* (**B**), AnStD7L1 salivary D7 from *An*. *stephensi* (**C**), and AeD7 salivary D7 from *Ae*. *aegypti* (**D**). The C-terminal domains are oriented in the same view by superposition, and key helical elements from each are labeled. The proteins show a continuum of increasing complexity and size from left to right, with the most complex, AeD7 containing a true internal binding pocket which contains a bound norepinephrine molecule having carbon atoms colored light grey, and nitrogen colored blue.

The C-terminal domain of ABI15936 shows a clear structural relationship to mosquito long-form D7s but the protein does not contain the interior binding pocket known to accommodate biogenic amine ligands in the long-form D7 AeD7 from *Ae*. *aegypti* and the short-form D7 protein D7r4 from *An*. *gambiae* (Fig. 8). Its positional relationship with the N-terminal domain is almost identical to the mosquito long-form D7s as well as mJHBP, a hemolymph D7-like juvenile hormone (JH)-binding protein from mosquitoes, and the domain interface contains several conserved interactions^15^. Comparison of ABI15936 with AeD7 shows that the region connecting helices α9 and α10 is truncated relative to AeD7, eliminating the equivalent of α-helix B2 of AeD7 that makes up one side of the ligand binding pocket. Additionally, the peptide chain terminates at Tyr 230, resulting in loss of the terminal helix H2 of AeD7, which forms a second side of the binding pocket. The *An*. *stephensi* long-form D7 AnStD7L1 and *Ae*. *aegypti* mJHBP also do not bind biogenic amines, and their crystal structures show rearrangement in the binding pocket region of the C-terminal domain related to the loss of α-helix B2 and changes in the positioning of the terminal α-helix that explain the absence of this function. However, the changes are not related to those seen in ABI15936 and involve rearrangements of structural elements rather than the absence of significant stretches of peptide sequence.

## Discussion

The complex salivary secretions of blood-feeding arthropods arise from recruitment of various types of housekeeping genes into the salivary transcriptome followed by modification to perform novel functions in the host environment during feeding. Multiple independent evolutions of the blood feeding habit have led to entirely different sets of protein families acquiring functions related to feeding in different lineages of blood feeders. This being the case, the independent evolution of blood feeding in the Culicomorpha and the Psychodidae has given rise to salivary secretions that are largely distinct in protein composition. Orthologous relationships of salivary proteins in the two groups are difficult to ascertain, and in most cases, important salivary functions are performed by proteins in completely different structural families. An example of this type of structural diversity would be the apyrase enzymes that hydrolyze the platelet activation agonist adenosine diphosphate. Potent apyrases exist in mosquitoes and sand flies, but the mosquito enzymes belong to the 5′-nucleotidase family, while the sand fly enzyme has a 5-bladed propeller structure that was later found in human apyrase^16,17^. A second example of divergent structures performing identical functions is the group of proteins responsible for scavenging of the biogenic amines histamine, serotonin and norepinephrine that are released by mast cells, platelets and peripheral nerve tissue on wounding of the host. These compounds are platelet activation agonists, vasoconstrictors, mediators of vascular permeability and inducers of pain and itching in the skin. In mosquitoes, biogenic amines are sequestered by all-helical short and long-form D7 proteins, while in sand flies they are bound by members of the 6-bladed β-propeller “yellow” protein family which is expressed throughout the Insecta, but outside the salivary gland serves functions in cuticle melanization and development^18^.

In this study we show that the long-form D7 proteins from sand flies are functionally like their counterparts in mosquito saliva and share a conserved structure. The N-terminal domain is especially like that of the mosquito forms with many key residues in the binding pocket being conserved. The C-terminal domain resembles that of the mosquito proteins but is missing major elements of the putative ligand binding pocket and does not bind small molecule ligands. Consistent with these structural observations, the sand fly proteins were found to perform the same anti-inflammatory and anti-platelet functions as their mosquito counterparts through the binding of cysteinyl leukotrienes and TXA_2_ in the N-terminal domain, but do not bind biogenic amines, as this function is performed by the yellow proteins.

The similarity of the long-form D7s, and especially the eicosanoid-binding N-terminal domains in mosquito and sand fly saliva, suggest that they have evolved from the same, or a very similar two-domain common ancestor, but the independent evolution of blood feeding in the two groups implies that selectivity for vertebrate eicosanoid ligands must have evolved independently. This is supported by the differences in exon structure in the mosquito and sand fly D7 genes. The molecular progenitor of the salivary D7 proteins is not known, but recently, mJHBP a non-salivary two-domain D7-like protein has been identified throughout the mosquito family and is the first protein of this type synthesized outside of the salivary gland^15^. This protein was found to circulate in the hemolymph of male and female adults and specifically binds JH at a comparable site in the N-terminal domain of the protein. The protein binds JH in the N-terminal domain pocket, while the terminal helix α13 of the C-terminal domain forms a cap-like structure that buries the ligand. However, no orthologs of this protein have been found in insects outside of the mosquito family, including the sand flies, making it unlikely to serve as a direct progenitor of the salivary D7s in both groups. Interestingly, the intron-exon structure of this gene differs from both the mosquito and sand fly salivary D7 genes.

Numerous single domain odorant binding protein family members occur in phlebotomine saliva, but other than the long form D7 proteins, these show a low degree of similarity to short or long form D7 proteins from Culicomorpha at the level of ligand binding pocket structure. In previous studies, single-domain sand fly OBP proteins from the saliva have been characterized, and do not function as small-molecule-binding proteins. PdSP15 from *P*. *duboscqi* is a 15 kDa OBP that binds polyphosphate and heparin and inhibits the contact pathway of coagulation, whose activation results in production of the proinflammatory effector bradykinin^19^. SALO, a protein closely related to PdSP15 from the new world species *Lutzomyia longipalpis* inhibits the classical pathway of complement activation by binding protein complexes involved in the initiation of complement activation^20,21^. The structures of these proteins show a typical single-domain OBP fold, but do not contain an internal binding pocket, and exhibit little detailed similarity to the short-form D7 proteins of mosquitoes. It appears that ancestors of Culicomorpha and Psychodidae may have independently recruited OBP genes into the salivary transcriptome and adapted them to perform different salivary functions. This is not particularly surprising since some protein families are particularly well-suited to salivary functions such as scavenging of small molecules, as demonstrated by the fact that they have evolved independently in widely disparate taxa. The most obvious example is the radiation of salivary lipocalins in ticks and triatomine bugs^22–24^. The lipocalins have been recruited into the saliva of the two groups independently but serve the same primary function of biogenic amine and eicosanoid binding in both. Structural analyses show that the ligand binding pockets of these proteins are not similar, and the binding modes of the ligands differ. Invoking convergent evolution is obvious in these cases, but comparison of the long-form D7 proteins from mosquitoes and sand flies reveal a high degree of conservation of binding pocket residues and an apparently similar ligand binding mode.

The diverse array of host processes potently affected by vector salivary proteins bestows them with potential for development as therapeutic agents. The dis-regulation of coagulation, platelet activation, immunity and inflammatory responses is responsible for many human diseases. Additionally, many of these blood feeders transmit serious parasitic, bacterial and viral diseases and saliva has been increasingly appreciated to modulate the host environment at the point of pathogen transfer, and in some cases to modify the pathogen, making it more infectious^4^. Inhibitors of various points of the coagulation cascade, of platelet activation, complement activation and inflammation have been studied for their potential usefulness in fighting maladies ranging from abnormal thrombosis to cancer^25–27^. Sand fly salivary proteins have played a particularly important role in the investigation of salivary proteins as vaccine antigens targeting pathogen transmission, in this case parasites of the genus *Leishmania*^3,28^. Understanding of the roles played by these proteins in disease transmission continues to grow, and clearly, their evolutionary diversification has increased the ways in which they can influence host physiology.

## Materials and Methods

### Protein expression and purification

*P*. *papatasi* AGE83092 (GI:449060611) and *P*. *duboscqi* ABI15936 (GI:112361959) long-form (two-domain) D7 DNA coding sequences were synthesized (Bio-Basic) lacking the signal sequence and cloned into the *E*. *coli* expression vector pET17b^12^. Proteins were expressed in *E*. *coli* BL21 (DE3) pLysS by induction with 1 mM IPTG for 3 hours at 37 °C. Inclusion bodies were either washed with 1% Triton X-100 in 10 mM Tris-HCl pH 7.4, 150 mM NaCl or washed with the detergent-free buffer. The inclusion bodies were dissolved in 6 M guanidine HCl, 20 mM Tris-HCl pH 8.0 containing 10 mM dithiothreitol and stirred for 30 minutes at room temperature. The guanidine solution was then added dropwise to 4 L of 20 mM CAPS pH 10, 400 mM arginine (AGE83092) or 20 mM Tris pH 8.5, 300 mM arginine, 1 mM cystamine (ABI15936). The refolded AGE83092 was extensively dialyzed against 20 mM Tris-HCl pH 7.5, 150 mM NaCl and 10 mM NaPO_4_ pH 6.0 and concentrated by ultrafiltration using a 10 kDa MW cutoff cellulose membrane (Milipore, USA). ABI15936 was concentrated by tangential flow ultrafiltration using a 10 kDa cutoff filter (Milipore, USA). For AGE83092 purification was performed using ion exchange chromatography on SP Sepharose followed by gel filtration on Sephacryl S-200. Concentrated ABI15936 was first separated by gel filtration on Sephacryl S-100. The pooled fractions were then diluted with 4 M ammonium sulfate in 20 mM sodium phosphate pH 7.4, applied to phenyl Sepharose, and the protein was eluted with a gradient of ammonium sulfate from 1.8 M to 0 M in sodium phosphate buffer. Purified proteins were concentrated using spin filters and the buffers were changed to 10 mM Tris pH 7.5. The purity of both proteins was assessed by SDS-PAGE with Coomassie blue staining. The selenomethionine derivative of ABI15936 protein was produced in the auxotrophic *E*. *coli* B834 BL21 (DE3) pLysS in SelenoMet media (Molecular Dimensions, USA) by induction with 1 mM IPTG also for 3 hours at 37 °C and purified as described above.

### Isothermal titration calorimetry

Proteins were diluted in 20 mM tris pH 7.4, 150 mM NaCl and degassed. Leukotrienes and U46619 in ethanol and methyl acetate, respectively, were dried under a stream of nitrogen and solubilized in 20 mM tris pH 7.4, 150 mM NaCl. These were sonicated for 10 minutes in a water bath sonicator and degassed before use. Biogenic amines were dissolved in the same buffer and degassed. Heats were measured with a MicroCal VP-ITC calorimeter (Malvern, United Kingdom) using 10 μl ligand injections over periods of 20 seconds with 300 seconds spacing the injections. The measured heats were integrated and fit to a single-site binding model using the MicroCal Origin software.

### Platelet aggregation measurements

Platelet-rich plasma (100 μL) collected from healthy donors (NIH Blood Bank) was combined with 200 μL Tyrode buffer (5 mM HEPES pH 7.4, 137 mM NaCl, 2 mM KCl, 1 mM MgCl_2_, 12 mM NaHCO_3_, 0.3 mM NaH_2_PO4, 5.5 mM glucose) and incubated with stirring at 37 °C in aggregometer (Chrono-log Corp, PA, USA). Before activation with native collagen fibrils (type I) from equine tendons (Chrono-log Corp, PA, USA) or the thromboxane A_2_ analog U46619, platelets were incubated with either the D7 proteins or buffer. Aggregation results were then recorded in duplicate as the change in transmittance of the platelet suspension.

### Protein crystallization

ABI15936 was crystallized using the hanging drop vapor diffusion method in 0.1 M MES pH 6.0, 20% w/v Polyethylene glycol 6000. After growth, crystals were flash cooled in liquid nitrogen in 0.1 M MES pH 6.0, 30% w/v polyethylene glycol 6000, 15% glycerol.

### Data collection and structure solution

Diffraction data were collected on beamline 22-ID of the Southeast Regional Collaborative Access Team (SER CAT) at the Advanced Photon Source, Argonne National Laboratory. The data were processed using HKL2000 or XDS and the structure of ABI15936 was solved by single anomalous diffraction (SAD) methods using Phenix Autosolve^29–31^. Two molecules of ABI15936 were contained in the asymmetric unit, and the model was largely built using the Phenix autobuilding routine and was completed manually using Coot^32^. Ligand electron density was evaluated using weighted Fo-Fc omit maps calculated using the Polder maps routine in Phenix^30^. Refinement and rebuilding were performed with phenix.refine with application of a TLS model and in Coot. The statistics for structure solution, model-building and refinement are shown in Table 1.

## Supplementary information

Figure S1

## Data Availability

The coordinates and structure factors have been deposited in the Protein Data Bank under the accession numbers 6MT7 (selenomethionine derivative) and 6MTF (wild type protein).

## References

[1] Ribeiro JM (1995). Blood-feeding arthropods: live syringes or invertebrate pharmacologists?. Infect Agents Dis.

[2] Kamhawi S, Aslan H, Valenzuela JG (2014). Vector saliva in vaccines for visceral leishmaniasis: a brief encounter of high consequence?. Front Public Health.

[3] Reed SG, Coler RN, Mondal D, Kamhawi S, Valenzuela JG (2016). Leishmania vaccine development: exploiting the host-vector-parasite interface. Expert Rev Vaccines.

[4] Pingen M, Schmid MA, Harris E, McKimmie CS (2017). Mosquito Biting Modulates Skin Response to Virus Infection. Trends Parasitol.

[5] Arca B, Lombardo F, Struchiner CJ, Ribeiro JM (2017). Anopheline salivary protein genes and gene families: an evolutionary overview after the whole genome sequence of sixteen Anopheles species. BMC Genomics.

[6] Ribeiro JM, Mans BJ, Arca B (2010). An insight into the sialome of blood-feeding Nematocera. Insect Biochem Mol Biol.

[7] Calvo E, Mans BJ, Andersen JF, Ribeiro JM (2006). Function and evolution of a mosquito salivary protein family. J Biol Chem.

[8] Calvo E, Mans BJ, Ribeiro JM, Andersen JF (2009). Multifunctionality and mechanism of ligand binding in a mosquito antiinflammatory protein. Proc Natl Acad Sci USA.

[9] Alvarenga PH (2010). The function and three-dimensional structure of a thromboxane A2/cysteinyl leukotriene-binding protein from the saliva of a mosquito vector of the malaria parasite. PLoS Biol.

[10] Abdeladhim M (2012). Updating the salivary gland transcriptome of Phlebotomus papatasi (Tunisian strain): the search for sand fly-secreted immunogenic proteins for humans. PLoS One.

[11] Martin-Martin I, Molina R, Jimenez M (2013). Molecular and immunogenic properties of apyrase SP01B and D7-related SP04 recombinant salivary proteins of Phlebotomus perniciosus from Madrid, Spain. Biomed Res Int.

[12] Giraldo-Calderon GI (2015). VectorBase: an updated bioinformatics resource for invertebrate vectors and other organisms related with human diseases. Nucleic Acids Res.

[13] Kato H (2006). High degree of conservancy among secreted salivary gland proteins from two geographically distant Phlebotomus duboscqi sandflies populations (Mali and Kenya). BMC Genomics.

[14] Abdeladhim M (2016). Molecular Diversity between Salivary Proteins from New World and Old World Sand Flies with Emphasis on Bichromomyia olmeca, the Sand Fly Vector of Leishmania mexicana in Mesoamerica. PLoS Negl Trop Dis.

[15] Kim IH (2017). A mosquito hemolymph odorant-binding protein family member specifically binds juvenile hormone. J Biol Chem.

[16] Champagne DE, Smartt CT, Ribeiro JM, James AA (1995). The salivary gland-specific apyrase of the mosquito Aedes aegypti is a member of the 5’-nucleotidase family. Proc Natl Acad Sci USA.

[17] Valenzuela JG, Belkaid Y, Rowton E, Ribeiro JM (2001). The salivary apyrase of the blood-sucking sand fly Phlebotomus papatasi belongs to the novel Cimex family of apyrases. J Exp Biol.

[18] Xu X (2011). Structure and function of a “yellow” protein from saliva of the sand fly Lutzomyia longipalpis that confers protective immunity against Leishmania major infection. J Biol Chem.

[19] Alvarenga PH (2013). Novel family of insect salivary inhibitors blocks contact pathway activation by binding to polyphosphate, heparin, and dextran sulfate. Arterioscler Thromb Vasc Biol.

[20] Asojo OA (2017). Structure of SALO, a leishmaniasis vaccine candidate from the sand fly Lutzomyia longipalpis. PLoS Negl Trop Dis.

[21] Ferreira VP (2016). SALO, a novel classical pathway complement inhibitor from saliva of the sand fly Lutzomyia longipalpis. Sci Rep.

[22] Francischetti IM, Andersen JF, Ribeiro JM (2002). Biochemical and functional characterization of recombinant Rhodnius prolixus platelet aggregation inhibitor 1 as a novel lipocalin with high affinity for adenosine diphosphate and other adenine nucleotides. Biochemistry.

[23] Mans BJ, Ribeiro JM, Andersen JF (2008). Structure, function, and evolution of biogenic amine-binding proteins in soft ticks. J Biol Chem.

[24] Xu X, Chang BW, Mans BJ, Ribeiro JM, Andersen JF (2013). Structure and ligand-binding properties of the biogenic amine-binding protein from the saliva of a blood-feeding insect vector of Trypanosoma cruzi. Acta Crystallogr D Biol Crystallogr.

[25] Carneiro-Lobo TC (2009). Ixolaris, a tissue factor inhibitor, blocks primary tumor growth and angiogenesis in a glioblastoma model. J Thromb Haemost.

[26] Decrem Y (2009). Ir-CPI, a coagulation contact phase inhibitor from the tick Ixodes ricinus, inhibits thrombus formation without impairing hemostasis. J Exp Med.

[27] Waxman L, Smith DE, Arcuri KE, Vlasuk GP (1990). Tick anticoagulant peptide (TAP) is a novel inhibitor of blood coagulation factor Xa. Science.

[28] Oliveira F (2015). A sand fly salivary protein vaccine shows efficacy against vector-transmitted cutaneous leishmaniasis in nonhuman primates. Sci Transl Med.

[29] Otwinowski Z, Minor W (1997). Processing of X-ray diffraction data collected in oscillation mode. Methods Enzymol..

[30] Adams PD (2010). PHENIX: a comprehensive Python-based system for macromolecular structure solution. Acta Crystallogr D Biol Crystallogr.

[31] Kabsch W (2010). Xds. Acta Crystallogr D Biol Crystallogr.

[32] Emsley P, Cowtan K (2004). Coot: model-building tools for molecular graphics. Acta Crystallogr D Biol Crystallogr.

