# Supplementary material for: Functional and structural similarities of D7 proteins in the independently-evolved salivary secretions of sand flies and mosquitoes
Source: Sci Rep. 2019 Mar 29;9:5340. doi: 10.1038/s41598-019-41848-0 (PMC6440969; doi:10.1038/s41598-019-41848-0)
Supplement: Supplementary file 1 — Figure S1 [file 41598_2019_41848_MOESM1_ESM.pdf]

Functional and structural similarities of D7 proteins in the independently-evolved salivary secretions of sand flies and mosquitoes.

Willy Jablonka, Il Hwan Kim, Patricia H. Alvarenga, Jesus G. Valenzuela, Jose´ M. C. Ribeiro, John F. Andersen*

The Laboratory of Malaria and Vector Research, NIAID, National Institutes of Health, Rockville, Maryland 20852 USA.

*Correspondence should be addressed to JFA E-mail: jandersen@nih.gov

Key words: Salivary gland, crystal structure, *Phlebotomus*

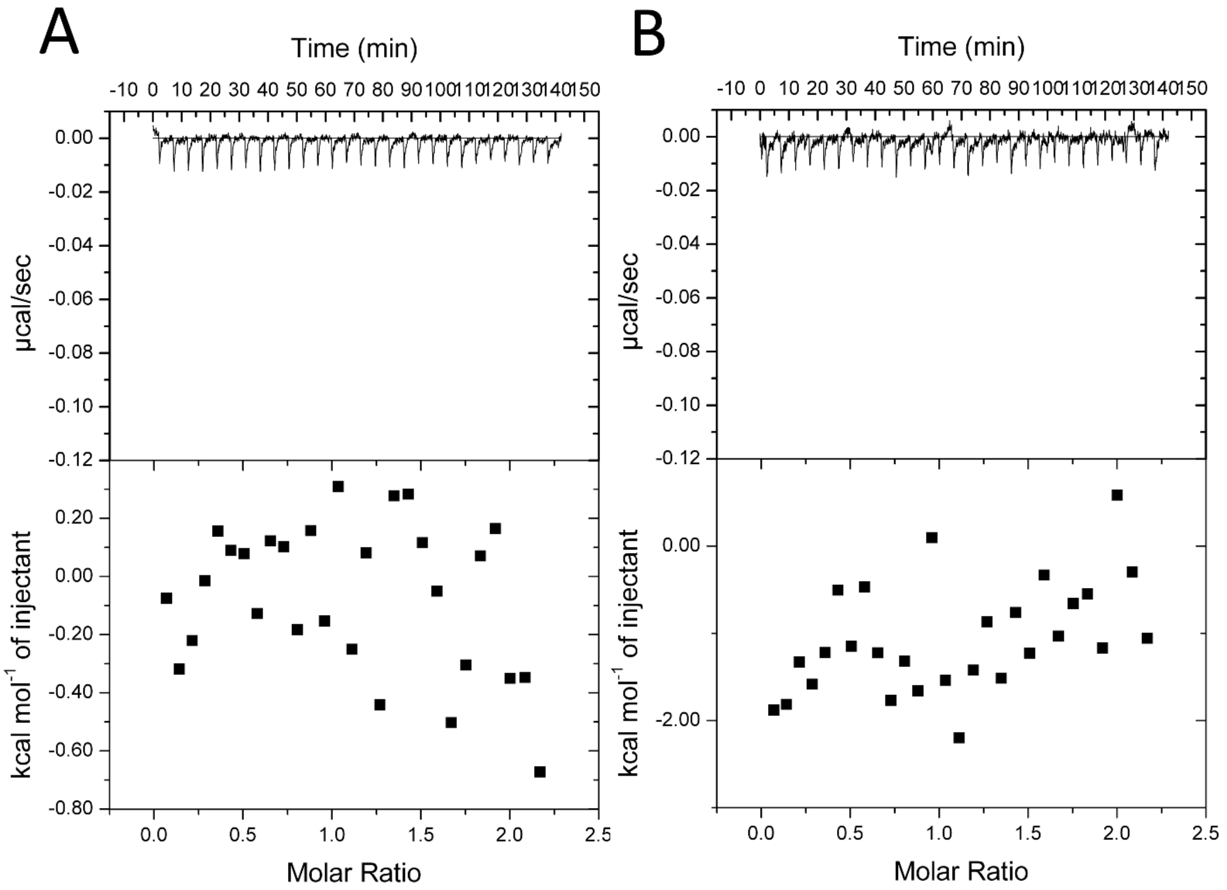

Fig. S1. ABI15936 does not bind histamine or serotonin. A solution of *P. duboscqi* ABI15936 (5 μM) was titrated with 50 μM of histamine (A) or serotonin (B) using serial 10 μL injections spaced by 300 seconds intervals. Heats were recorded on a VP-ITC MicroCalorimeter (Malvern, United Kingdom).
